# Infarction and Rupture of the Heart

**Published:** 1907-10-05

**Authors:** 


### Illustrative Cases.

### INFARCTION AND RUPTURE OF THE HEART.

Rupture of the heart, if we exclude holes made in the organ by bullets, swords, and so forth, is most commonly due to the effects of syphilis. A gummatous infiltration of the cardiac muscle heals with the formation of a fibrous scar, which may or may not stretch so as to form a cardiac aneurysm, but which in any case is a weak spot in the wall, and, as the result of some particularly strenuous effort, may rupture, the result being sudden death. These syphilitic scars may occur anywhere in the heart, but those which rupture are nearly always near the apex of the left ventricle.

The following case is not one of this sort, but is an example of cardiac infarction due to the giving way of a very atheromatous coronary artery in an old man; the infarction occurred during sleep; it was not embolic; the symptoms of the hæmorrhage into the cardiac wall are very clearly demarcated from those of the subsequent rupture of the infarcted area, which occurred eleven hours later, and caused sudden death. The diagnosis was confirmed by autopsy.

A male patient, aged 71, was under treatment for severe and generalised eczema. He had not had syphilis, nor had he been intemperate in his use of either alcohol or tobacco. He looked a hale and hearty man, notwithstanding his years. There was no albuminuria, and his lungs and heart exhibited no abnormal physical signs. The eyes were normal to ophthalmoscopic examination, and but for his eczema he seemed quite well. One day, finding time hang heavy on his hands, he went to bed about half-an-hour after his midday meal; when he had slept about an hour he was suddenly awakened by an excruciating pain in the præcordial region, rapidly followed by vomiting. He was seen very shortly afterwards. His face was then pale and drawn, his extremities cold and clammy, and his forehead bathed with perspiration. There was only slight dyspnœa. The pulse was small, irregular in volume, and sometimes intermittent. Auscultation of the heart revealed no bruit, but a lack of clearness of the sounds and a certain degree of arrhythmia. Nothing abnormal could be heard in the lungs.

The pain, though intense, was not very localised; it was referred to the third, fourth, fifth, and sixth intercostal spaces, both in front and behind, without any one spot being more painful than another. There was no radiation of the pain towards the left shoulder nor down the left arm. The patient, moreover, far from keeping that immobility which is such a striking feature of angina pectoris, was rolling about upon the bed. He was quite clear-headed, and replied freely to any questions that were put to him.

A mustard plaster to the præcordia and a subcutaneous injection of caffein gave rapid relief to the intensity of the symptoms, but failed to relieve them entirely. Seven hours later he was still suffering, but less; the pulse was stronger, but the heart was still irregular in rhythm. A fresh symptom now developed—namely, a short dry cough, without expectoration. Fine râles could be heard at the bases of the lungs. The initial vomiting did not recur. Three hours later—that is to say, ten hours from the onset—the dyspnœa increased considerably, though there was little or no cyanosis. The countenance remained pale, the præcordial pain was still present, and the heart and pulse showed the same features as before.

Expectoration of frothy serous fluid now became profuse, and there were widespread râles extending up from the bases of the lungs, and suggesting acute pulmonary œdema. It seemed wise to venesect; the patient was relieved by this, and seemed to be much better, when, at eleven hours from the onset, there was a sudden syncope and the man was dead.

The post-mortem examination showed turgid œdematous lungs, healthy kidneys and abdominal viscera, and the following cardiac condition:

The pericardium contained $6\frac{1}{2}$ oz. of dark blood clot. The heart itself weighed $11\frac{3}{4}$ oz., and exhibited neither excess of fat nor any obvious fibroid changes. On the front of the left ventricle, near the apex, there was a linear orifice, with comparatively sharp edges, looking as if the hole might have been made by a cutting instrument. It was $\frac{3}{4}$ in. in length, and lay in the long axis of the heart. It formed the centre of a hæmorrhagic area the size of a florin, and fragments of dark clot were projecting between the lips of the hole. There was no generalised degeneration of the myocardium; on cutting open the left ventricle it was found that the whole of the muscle round the hole was converted into one large hæmorrhagic infarct throughout the whole thickness of the ventricle at this spot. The valves showed no more than the usual degree of atheroma; but on examining the coronary arteries it was found that whereas the posterior was moderately permeable in spite of extensive atheromatous deposits, the anterior was very nearly occluded by a ring of calcareous material at a point half-an-inch from its origin. The calcareous ring was itself half-an-inch in length, though above and below the ring the artery was much more patent. It seemed clear that there had been no embolism of either artery, but that the anterior vessel had either given way, or else had become plugged by thrombosis at the site of its greatest narrowing, the result being a hæmorrhagic infarct of the parts beyond. The infarction had occurred during sleep, and had caused the extreme precordial pain and the vomiting. The wall had remained intact for eleven hours, but on account of the progressive breaking up of the cardiac muscle fibres by the extravasated blood, together with acute local necrosis, such as always occurs in an infarct, the wall suddenly gave way eleven hours later, and caused the terminal syncope. The final rupture was, no doubt, accelerated by the dyspnœa which was due to the acute pulmonary œdema.

The condition is by no means common, but neither is it extremely rare in old people, though text-books say little or nothing about it. The subject has been much studied in France, and the following are summaries of the pathology and symptoms as given by Ranvier and by Robin respectively:

"Si la mort n'est pas instantanée, le sang s'infiltre en écartant les faisceaux musculaires et en donnant lieu à une hémorragie interstitielle diffuse. Si la déchirure ne va pas jusqu'a péricarde, il se fait un aneurysme diffus d'abord, qui peut ensuite se circonscrire; mais le plus souvent la déchirure se poursuit jusque dans le péricarde.

"Il semble que la rupture se fasse en plusieurs fois, à en juger par le développement d'accidents formidables, caractéristiques, qui s'amendment et laissent après eux un calme plus ou moins sensible.

"Après cette première atteinte, il y a une sorte d'atténuation des symptômes douloureux. Mais ce qui est tout à fait particulier et différencie ceci de l'angine de poitrine ordinaire, c'est que, la période aiguë une fois passée, ils ne disparissent pas, maise persistent sous une forme plus ou moins atténuée, jusqu'à l'accès terminal."

The case described may, therefore, be regarded as a very typical one of cardiac infarction from atheroma, and subsequent fatal rupture of the heart.

